# Criteria and Indicators for Centers of Clinical Excellence in Stroke Recovery and Rehabilitation: A Global Consensus Facilitated by ISRRA

**DOI:** 10.1177/15459683231222026

**Published:** 2024-01-11

**Authors:** Rachel C. Stockley, Marion F. Walker, Margit Alt Murphy, Noor Azah Abd Aziz, Philemon Amooba, Leonid Churliov, Amanda Farrin, Natalie A. Fini, Emma Ghaziani, Erin Godecke, Tania Gutierrez-Panchana, Jie Jia, Thoshenthri Kandasamy, Patrice Lindsay, John Solomon, Vincent Thijs, Tierney Tindall, Donna C. Tippett, Caroline Watkins, Elizabeth Lynch

**Affiliations:** 1 Stroke Research Team, School of Nursing and Midwifery, University of Central Lancashire, Preston, UK; 2 School of Medicine, University of Nottingham, Nottingham, UK; 3 Institute of Neuroscience and Physiology, Department of Clinical Neuroscience, Rehabilitation Medicine, Sahlgrenska Academy, University of Gothenburg, Gothenburg, Sweden; 4 Department of Family Medicine, Medical Faculty, National University of Malaysia (UKM), Bangi, Malaysia; 5 Department of Nursing, Kwame Nkrumah University of Science and Technology, Kumasi, Ghana; 6 Melbourne Medical School, The University of Melbourne, Melbourne, VIC, Australia; 7 Clinical Trials Research Unit, Leeds Institute of Clinical Trials Research, University of Leeds, Leeds, UK; 8 Physiotherapy Department, School of Health Sciences, The University of Melbourne, Melbourne, VIC, Australia; 9 Department of Physical and Occupational Therapy, Copenhagen University Hospital-Bispebjerg and Frederiksberg Hospital and Department of Brain and Spinal Cord Injury, Copenhagen University Hospital—Rigshospitalet, Copenhagen, Denmark; 10 School of Medical and Health Sciences, Edith Cowan University and Sir Charles Gairdner Osborne Park Health Care Group, Joondalup, WA, Australia; 11 Clinica Alemana Universidad del Desarrollo, Concepción, Chile; 12 Department of Rehabilitation, Huashan Hospital Fudan University, Shanghai, China; 13 Caring Futures Institute, Flinders University, Adelaide, SA, Australia; 14 Heart and Stroke Foundation of Canada, Toronto, ON, Canada; 15 Centre for Comprehensive Stroke Rehabilitation and Research, Department of Physiotherapy, Manipal College of Health Professions, Manipal Academy of Higher Education, Manipal, India; 16 Department of Medicine University of Melbourne, Department of Neurology Austin Health, Florey Institute of Neuroscience and Mental Health, Parkville, VIC, Australia; 17 Mental Health and Clinical Neurosciences, School of Medicine, University of Nottingham, Nottingham, UK; 18 Departments of Physical Medicine and Rehabilitation, Neurology, and Otolaryngology—Head and Neck Surgery, Johns Hopkins University, Baltimore, MD, USA

**Keywords:** consensus, leadership, stroke, rehabilitation, organizational culture, delivery of healthcare

## Abstract

**Background:**

The aim of the International Stroke Recovery and Rehabilitation Alliance is to create a world where worldwide collaboration brings major breakthroughs for the millions of people living with stroke. A key pillar of this work is to define globally relevant criteria for centers that aspire to deliver excellent clinical rehabilitation and generate exceptional outcomes for patients.

**Objectives:**

This paper presents consensus work conducted with an international group of expert stroke recovery and rehabilitation researchers, clinicians, and people living with stroke to identify and define criteria and measurable indicators for Centers of Clinical Excellence (CoCE) in stroke recovery and rehabilitation. These were intentionally developed to be ambitious and internationally relevant, regardless of a country’s development or income status, to drive global improvement in stroke services.

**Methods:**

Criteria and specific measurable indicators for CoCE were collaboratively developed by an international panel of stroke recovery and rehabilitation experts from 10 countries and consumer groups from 5 countries.

**Results:**

The criteria and associated indicators, ranked in order of importance, focused upon (i) optimal outcome, (ii) research culture, (iii) working collaboratively with people living with stroke, (iv) knowledge exchange, (v) leadership, (vi) education, and (vii) advocacy. Work is currently underway to user-test the criteria and indicators in 14 rehabilitation centers in 10 different countries.

**Conclusions:**

We anticipate that use of the criteria and indicators could support individual organizations to further develop their services and, more widely, provide a mechanism by which clinical excellence can be articulated and shared to generate global improvements in stroke care.

## Introduction

The Stroke Recovery and Rehabilitation Roundtables provided a collaborative forum for preclinical and clinical stroke researchers to work alongside methodologists, consumer groups, statisticians, and funders to accelerate identification and implementation of effective treatments to improve stroke recovery and rehabilitation.^1^ Building on this work, the International Stroke Recovery and Rehabilitation Alliance (ISRRA) was established to create a world where global collaboration brings major breakthroughs for people living with stroke. Specifically, ISRRA seeks to be a “go-to” place for researchers interested in recovery and rehabilitation, to identify new targets for consensus building and funding priorities for research.^2^

In a facilitated meeting attended by 60 world leading stroke experts and members of ISRRA in 2018,^2^ one of the key pillars of work identified to advance the field of stroke recovery and rehabilitation was to generate globally applicable criteria for Centers of Clinical Excellence (CoCE). It was envisaged that defining clinical excellence in stroke recovery and rehabilitation could guide service development, focus research priorities, and facilitate global networks to transform the standard of stroke care across the world.

In wider literature, centers of excellence are characterized by the use of innovative methods, a collaborative approach, and high-quality service^3-6^ that produce exceptional outcomes and significant scientific, political, economic, or societal impacts.^4^ It is widely agreed that CoCE should demonstrate expertise in a specific area to enable delivery of comprehensive interdisciplinary care that optimizes patients’ outcomes.^5^ In stroke, many models, standards, and measures have been developed to reduce variability in care and demonstrate clinical effectiveness. These include identification of optimal models of acute stroke care in high income countries,^7^ key metrics of clinical performance,^8,9^ and evidence-based national guidelines.^10,11^ These outputs typically articulate the interventions that should be provided, by when and by whom^9,10,12^ and are clearly valuable to improve clinical practice. However, they focus upon the *products* of excellent care and do not articulate the vital *processes* necessary to embed excellence in stroke care.^5,6^ These processes are much less clear and there are no globally applicable criteria that consider the key features of clinical centers that deliver excellent stroke rehabilitation. This means that stroke services cannot identify the properties, approaches, and culture that are likely to be necessary to provide excellent care in their setting.

Despite a proliferation of organizations that apply clinical excellence monikers to their services^3,5,6^ there is not a recognized process by which CoCE can be identified, developed, or measured. The aim of this work was to develop globally-relevant criteria to define CoCE in stroke recovery and rehabilitation and to generate measurable indicators for each criterion that can be used by centers to assess the quality of their current services. These criteria and indicators must be sufficiently broad to enable tailoring for different resource and geographical settings, but appropriately specific to ensure clarity, transparency, and usability. This work constitutes an important first step in realizing an ambitious vision to drive up the quality of global stroke care. Used in concert with metrics of clinical performance and national guidelines, these criteria and indicators of CoCE could identify the components that are likely to engender excellence and, by judging performance, recognize excellence that can be shared with other centers through ISRRA and others’ global networks.

## Methods

**Figure 1.**
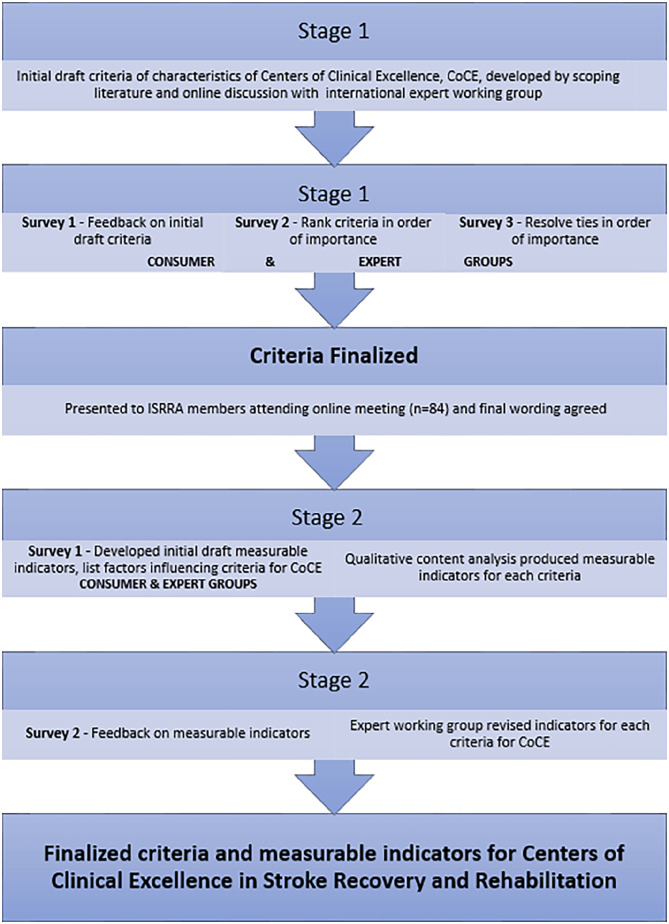
Stages in development of criteria and measurable indicators for CoCE.

An international multi-disciplinary expert working group was convened in 2020. ISRRA members self-nominated or were purposively invited to join the CoCE working group so there was representation from diverse geographic and socioeconomic areas, career stage, and professional backgrounds (including clinical and methodological expertise). Working group members were selected based upon their knowledge and extensive track record of contribution to stroke recovery and rehabilitation, experience of different global settings, and enthusiasm for international stroke service development. A structured multi-step procedure (shown in Figure 1) to identify and prioritize criteria and measurable indicators for CoCE was developed incorporating Keeney’s Value Focused Thinking methodology,^13^ which has been used successfully in previous international stroke consensus projects.^14^ People living with stroke (survivors and carers) were consulted at each stage through seeking feedback from consumer groups. These were purposively selected for consultation as they were longstanding, established well-functioning groups of many years standing with a diverse membership. They represented people from low-, middle-, and high-income settings with different healthcare models, and were identified by members of the expert working group as having extensive previous experience of providing critical and constructive feedback to stroke research. Within each group there was an open call for inclusiveness and representativeness to participate with this work.

### Stage 1: Developing and Defining the Criteria of CoCE

The expert working group met online to discuss factors that could contribute to clinical excellence in stroke recovery and rehabilitation and scoping of relevant literature was undertaken to identify definitions of clinical excellence in other health conditions. Through a series of online meetings, the expert group identified key areas that were perceived to influence excellence in stroke recovery and rehabilitation and began to refine and draft initial criteria for each, merging similar areas together where possible. These criteria were deliberately aspirational, aligning to ISRRA’s goal to bring about major breakthroughs for people living with stroke.

Three surveys (see Supplemental 1) were sent to all expert working group members. Survey 1 included open-ended questions about the purpose of identifying CoCE to gain knowledge from other clinical areas and feedback on the initial draft criteria. Survey 2 asked respondents to rank the relative importance of each criterion of clinical excellence. A structured process^15^ using a graph theory-based voting system was used to aggregate these rank-ordered lists wherein a directed graph, called the preference graph, was used to represent the patterns of ranking responses. Vertices of the graph represented the criteria ranked by the respondents, and directed edges corresponded to preferences between these criteria. This method of combining preference scores avoids inappropriate use of averaging. This approach was used in preference to other, more well-known approaches such as Delphi, to allow inclusion of a wide variety of items while also accounting for potential differences in the perceived importance of these items to different respondents.^13^ A third survey was required because, after Survey 2, 3 criteria were perceived to be equally important; Survey 3 asked respondents to rank the importance of these 3 criteria relative to each other.

Four consumer groups comprising people after stroke and their carers based in the UK, India, Malaysia, and Australia provided feedback on the initial and evolving criteria and participated in ranking the criteria in order of importance. Whilst surveys were in English, in areas where English was not the first language some members of the consumer groups spoke English and were able to assist in translation and interpretation of the groups’ responses. The groups’ facilitators were also able to help with culturally appropriate translations of particular words and phrases. Final wording of the criteria was collectively edited by the expert working group and these draft criteria for CoCE were presented to the consumer groups and to 84 ISRRA members in October 2020 for feedback, which was incorporated into the final criteria.

### Stage 2: Identification of Measurable Indicators

A second round of online discussions was held with the expert working group to identify measurable indicators for each criterion, followed by 2 surveys (Surveys 4 and 5). Survey 4 consisted of 3 open-ended questions for each criterion in which respondents were asked to generate the elements that defined the criterion and nominate barriers and enablers to realizing excellence in the criterion (21 questions in total, Supplemental 1). The survey was sent to members of the expert working group and an aphasia-friendly version of the survey was sent to consumer groups in the USA, Australia, UK, and Malaysia.

Responses to Survey 4 were analyzed using qualitative content analysis by 3 authors (RCS, EL, and TK), using inductive coding to identify the common keywords and concepts. Responses regarding barriers and enablers were checked for additional elements that could be included to define the criteria. Data were further refined into measurable indicators, then checked for ambiguity, redundancy, and duplication. Survey 5 containing the draft list of indicators for each criterion was circulated to the expert working group and consumer groups. Feedback about whether all relevant concepts were presented and the clarity of the indicators (particularly from people for whom English was not their first language) was sought. This was used to refine and finalize measurable indicators for each of the criteria of CoCE in stroke recovery and rehabilitation.

## Results

The expert working group comprised 20 recovery and rehabilitation experts from 10 countries (Australia, Canada, Chile, China, Denmark, Ghana, India, Malaysia, Sweden, USA, and the UK). Members’ professions spanned acute neurology (n = 1) family medicine (n = 1), nursing (n = 2), methodological expertise (n = 2), occupational therapy (n = 2), physical therapy (n = 6), rehabilitation medicine (n = 4), and speech and language therapy (n = 2). Five consumer groups were included: the Australian Stroke Foundation’s Consumer Council; Nottingham Stroke Research Partnership, UK; National Stroke Association Malaysia; the community outreach program of Centre for Comprehensive Stroke Rehabilitation and Research, MAHE, Manipal India; and Snyder Center for Aphasia Life Enhancement, Maryland, USA.

### Criteria of CoCE

The expert working group defined a CoCE as comprising a network of linked services across the stroke pathway. A CoCE may or may not be at a single geographical site or discrete building and, in stroke services, may include both acute and follow-on community services. Inclusive, equitable principles, and the experiences of people living with stroke and carers were embedded within all criteria to ensure that CoCE serve diverse and multi-cultural communities.

**Figure 2.**
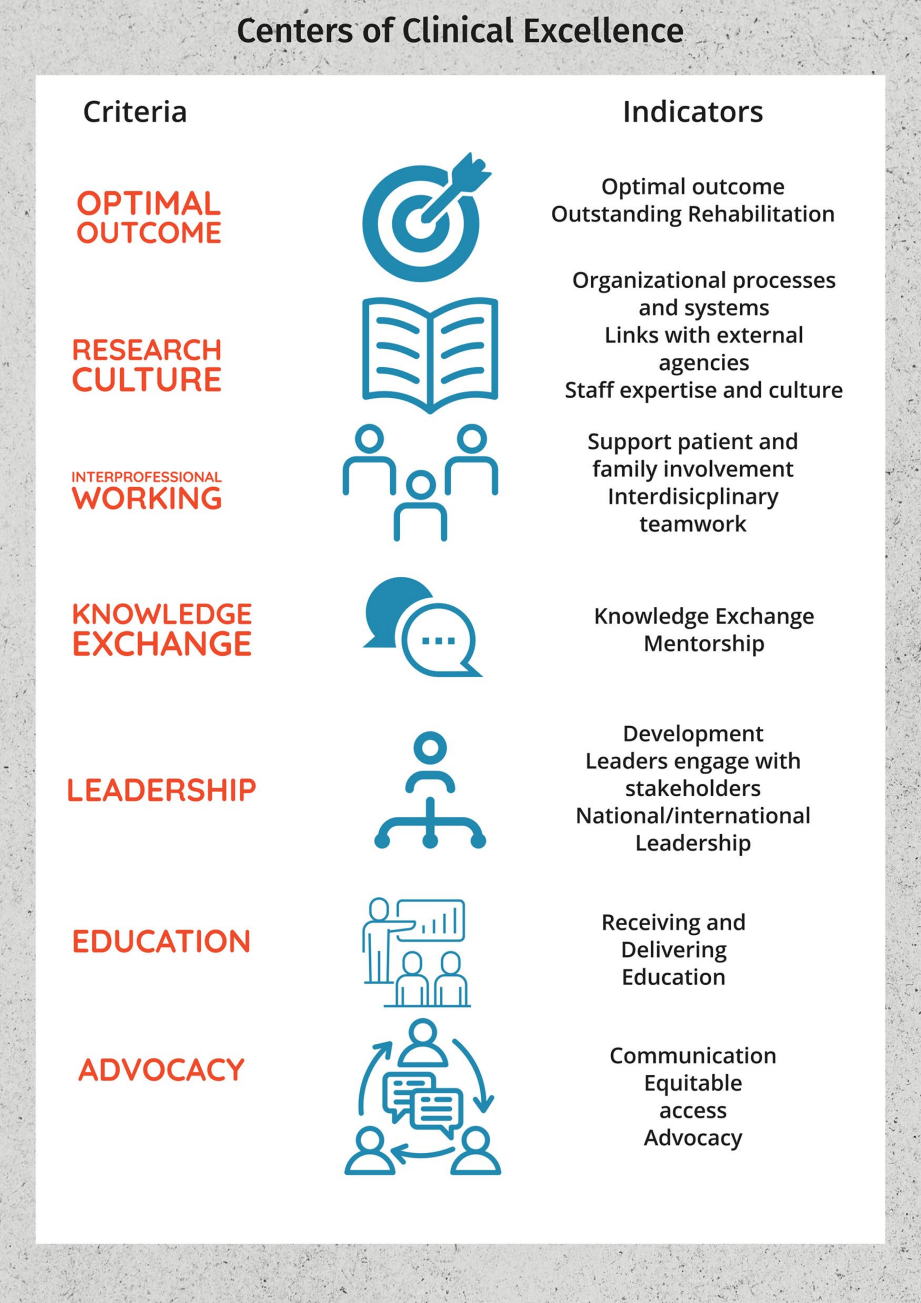
The 7 criteria and summary of measurable indicators for CoCE in stroke recovery and rehabilitation, ranked in order of importance.

Seven criteria were agreed and were ranked in order of importance (Figure 2 and Table 1).

**Table 1.** Table of Criteria and Measurable Indicators for Centers of Clinical Excellence (CoCE) in Stroke Recovery and Rehabilitation.

Criteria	Measurable indicators		
CoCE in stroke and recovery and rehabilitation:	Category	Indicator groups	Indicator sub-groups (where required)
1. Deliver outstanding rehabilitation to ensure optimal outcome (health, social, and wellbeing) for people living with stroke.	Optimal outcomes	Patient outcomes	Clinical/physiological measures
			Patient reported outcomes
			Patient reported experience
			Self-management skills
		Carer outcomes	Carer reported outcomes
			Carer reported experience
			Carer self-management skills
		Service outcomes	
	Deliver outstanding rehabilitation	Assessment of rehabilitation requirements	Comprehensive/holistic assessment
			Ongoing assessment at regular time points
		Rehabilitation interventions	Evidence-based 1. Time after stroke when rehabilitation started 2. Duration 3. Dose 4. Procedures/methods
			Addresses patient’s goals (tailored rehabilitation)
			Integrated delivery (minimize duplication between professionals/services)
		Coordinated ongoing care and support	
2. Have a strongly developed research culture, demonstrated by proactive national and international research collaborations, and translation of research into best clinical practice.	Organization-al processes and systems	Research elements in all job descriptions and role profiles	
		Organized initiatives to support positive research culture	Regular research activities for all staff, for example, journal clubs, training, or attending conferences
			Embedded quality improvement program Regular collection of outcome data
		Infrastructure and resources to support research activity	Allocated research time
			Systems to support high quality data collection
		A recognized pathway or strategy to implement research into practice	
	Formalized links with external agencies	Links with universities	
		Research collaborations with other national and international centers	
	Staff expertise and culture	Leading research, applying for and winning research funding	
		Research leadership from multiple professional groups	
		Broad methodological research knowledge across staff base (or access to skills/knowledge)	
3. Ensure inter-professional working and person-centered rehabilitation where colleagues, persons with stroke, and carers work together toward a common goal.	Organizations and systems to proactively support patient and family involvement in rehabilitation journey	Information provided routinely to patient and family about rehabilitation process and rehabilitation team	
		Collaborative goal setting process (goals agreed upon by team, patient, and family)	
		Regular opportunities between team, patient and family for 2-way information exchange	
		Shared decision-making between rehabilitation team, patients, and carers	
		Virtual communication available when indicated (eg, lockdowns and supporting remote services)	
		Processes to identify all key stakeholders in stroke rehabilitation within and beyond the center	
		Culturally safe care provision	
	Systems to support coordinated inter-professional teamwork	Regular opportunities for rehabilitation team to collaboratively review patient goals, progress, and plans	
		Input from each team member is respected and valued	
4. Exchange new knowledge and actively promote mentorship with National/International colleagues and people living with stroke to advance best practice.	Knowledge exchange	Collaborations with external organizations to exchange knowledge about best practice, for example, clinical practice groups and national and international rehabilitation groups	
		Protected time allocated for knowledge exchange activities, for example, networking	
		Opportunities for staff to participate in training using different modalities for knowledge exchange activities, for example, TED talk, social media, radio, and TV	
	Mentorship	Formal interdisciplinary mentorship program (eg, allocated mentors and mentees) for individual clinicians and people living with stroke	
		Formal mentorship program for clinical centers	
		Investment in mentorship training for mentors	
		Protected time for mentoring	
5. Have a shared strong ethical and value-based leadership, that inspires, motivates, and drives forward successful rehabilitation.	Development	Rehabilitation workforce development	Commitment to recruitment of the “best” staff (based on competency and experience)
			Processes to promote professional growth and development of staff
		Leadership development	Mechanisms to gain feedback to/about leaders and assess leadership, for example, 360° feedback, formal appraisals, and open door policies
			Investment in training and time to grow leaders (who are open minded, adaptive, inclusive, team focused, and knowledgeable)
			Systems to support staff to take up global leadership roles (eg, editorial boards and committees)
	Leaders engaging with key stakeholders	Engagement of leadership with patients and carers	
		Leadership actively promotes delivery of successful rehabilitation	
	National/inter-national leadership	Representation on influential national/international groups and professional bodies	
6. Use their specialist knowledge to provide continuous high-quality education to people with stroke, carers, staff, and the general public (Formal education such as In-house training, Masters Courses, Conference Presentations, and Public Lectures).	Receiving education	Pathways for staff to gain higher-degree qualifications including Master’s and PhD	
		Onsite educational opportunities, for example, inhouse training	
		Support for off-site education, for example, sponsored workplace visits, conference scholarships, sabbaticals to other centers	
	Delivering education	Delivering conference presentations and in-services to health professionals	
		Providing education to stroke survivors and carers, and the public	
7. Advocate and promote equitable access and optimal delivery of stroke rehabilitation services and funding for innovative research	Processes that facilitate ongoing communication with key stakeholders	—	
	Equitable access of stroke rehabilitation	Systems to promote equitable access	
		Processes to monitor access	
		Processes to improve access if problems identified	
	Regular advocacy and outreach activities	For access to stroke rehabilitation services	
		For innovative research	

Each criterion and the measurable indicators are summarized below in order of perceived importance and presented in detail in Table 1. Each criterion is accompanied by a short rationale and examples of practical application.

1. CoCE *in Stroke Rehabilitation and Recovery deliver outstanding rehabilitation to ensure optimal outcomes (health, social, and wellbeing) for people living with stroke.*

Optimal outcome recognizes that recovery and wellbeing are influenced by a range of factors alongside physical and mental improvement after stroke, including emotional and social issues. Measurable indicators were grouped to define optimal outcomes (patient, carer, and service), and the delivery of outstanding rehabilitation (assessment, rehabilitation interventions, and coordinated ongoing care and support). Excellent clinical services should utilize robust processes to measure and understand their impact upon both health and holistic wellbeing and ensure that the voices of people living with stroke, where cognition allows, and their carers are central to their evaluations.

2. CoCE *in Stroke Rehabilitation and Recovery have a strongly developed research culture, demonstrated by proactive national and international research collaborations and translation of research into best clinical practice.*

A developed research culture encompasses a range of activities such as proactive research collaborations, local research activity and implementation of research evidence into practice. Groups of measurable indicators to demonstrate a positive research culture included overt recognition of research in organizational processes and systems, formalized links with external, research active agencies and staff research expertise and culture.

The expert working group noted that, in practice, this is likely to require generic skills at the level of the organization, for instance in change management and knowledge translation, as well as supporting participation in, and undertaking, ethically-sound research.

3. CoCE *in Stroke Rehabilitation and Recovery ensure inter-professional working and person-centered rehabilitation where colleagues, persons with stroke and carers work together toward a common goal.*

It was recognized that clinical excellence is likely to be achieved when people living with stroke and their carers, work as equal partners with clinicians and other stakeholders toward a common goal. This requires robust processes that ensure people with stroke (if cognitively able) and their carers are actively and fully included in goal setting and decision-making. Measurable indicators were grouped to reflect the need for organization’s processes that proactively support the patient and their family to be involved in the rehabilitation journey and systems that enable coordinated inter-professional teamwork. Achieving clinical excellence was also likely to be dependent upon teams within health settings working together with others (eg, technology developers, engineers, charities, and leisure providers) and communicating effectively to deliver efficient, person-centered rehabilitation with seamless transitions in care.

4. CoCE *in Stroke Rehabilitation and Recovery exchange new knowledge and actively promote mentorship with National/International colleagues and people living with stroke to advance best practice.*

The importance of knowledge exchange to facilitate the sharing of best practice and learning to ensure high quality clinical practice that delivers optimal outcome after stroke was acknowledged. Measurable indicators centered on 2 areas: knowledge exchange with policy-makers, practice bodies and industry, nationally and internationally; and mentorship both between individuals (people living with stroke who are contributing to service improvement initiatives as well as clinicians) and clinical centers.

5. CoCE *in Stroke Rehabilitation and Recovery have a shared strong ethical and value-based leadership, that inspires, motivates, and drives forward successful rehabilitation.*

Leadership grounded in ethics and linked to organizational values was recognized to promote the delivery of clinical excellence. It was recognized that staff should be supported to consider how they work together and how they could improve team working. Whilst local leadership impacts the day-to-day activities of teams and individuals, higher-level leadership was deemed vital to ensure that the services are configured to support clinical excellence and can respond flexibly to changes in demand and direction in clinical practice. Measurable indicators for this criterion measured development of the workforce and leadership, engagement between stakeholders and leaders locally, nationally, and internationally.

6. CoCE *in Stroke Rehabilitation and Recovery use their specialist knowledge to provide continuous high-quality education to people with stroke, carers, staff, and the general public.*

Whilst education of the clinical team is recognized as key element to promote clinical excellence, it was noted that education initiatives should extend to people living with stroke, their carers, industries, and the wider public. Measurable indicators focused on staff opportunities to engage with education to improve their skills and knowledge and the delivery of education by the center (eg, public engagement, to stroke survivors and cares, and professional fora).

7. CoCE *in Stroke Rehabilitation and Recovery advocate and promote equitable access and optimal delivery of stroke rehabilitation services and funding for innovative research.*

A CoCE should actively support people living with stroke by working to ensure equitable access to acute stroke care and early rehabilitation, and by promoting innovative, cross-disciplinary research. Three groups of measurable indicators were developed: ongoing communication with key stakeholders, equitable access to stroke rehabilitation and advocacy and outreach services. It was acknowledged that these should empower all people interested in stroke services, including people with stroke and their carers, to shape current services, and generate the next breakthroughs in clinical care and stroke rehabilitation research.

## Discussion

To the best of our knowledge, our work is the first to define the key criteria and measurable indicators of CoCE in stroke recovery and rehabilitation and so constitutes an important first step in realizing ISRRA’s vision to improve global stroke care. Our criteria extend what is already available by reaching beyond what is expected toward what is ideal, to optimize holistic stroke recovery, and so have the potential to advance the field of stroke rehabilitation. The criteria and indicators were developed collaboratively and explicitly recognize that clinical excellence in stroke recovery and rehabilitation is likely to be a multi-faceted, emergent property of the systemic interactions between staff, people living with stroke, carers, industry partners, and organizational factors. Unlike previous work that has described excellence as a product,^4^ our criteria clearly recognize that a culture that fosters and supports excellence is vital and that clinical excellence is likely to require an iterative process of continuous improvement.

Use of the criteria and associated indicators provides a mechanism by which clinical excellence can be identified, described, and shared to generate global improvements in stroke care, organizational development and shape the culture required to deliver excellence.^4,5^ The criteria and indicators presented here have the potential to support organizations that aspire toward excellence to develop or refine their services, staff, and activities. Work is currently underway to user-test the criteria and indicators in 14 centers in 10 countries: Australia, Chile, China, Denmark, Ghana, India, Malaysia, Singapore, Sweden, and the UK. This will identify the data that could be collected to demonstrate performance for each of the criteria and enable us to characterize, and define, how excellence will be judged for each criterion. We anticipate that these indicators will complement but may overlap other metrics of quality stroke care,^9-12^ particularly clinical practice guidelines which form part (but not all) of the most important criterion identified (Criterion 1 *“Deliver outstanding rehabilitation”*). To address any overlap and following user-testing, we will map the data required to demonstrate achievement of excellence in the criteria against existing routine data collection processes to assess duplication. Inefficiencies in data collection will be minimized by aligning the finalized criteria and indicators with routinely collected data when this is appropriate, to reduce data collection burden.

Once finalized, ISRRA will ensure global dissemination of the criteria and indicators through its membership (which currently exceeds 500 global members), academic, and professional networks (eg, the World Stroke Organization, WSO and World Rehabilitation Alliance). We are currently exploring ways we can partner with others who seek to improve stroke care and rehabilitation to ensure this work has maximum reach and impact (eg, discussions are underway with the WSO). In keeping with the philosophy of ISRRA, the primary intent of this work is for global centers to use the criteria and indicators to guide their development toward excellence. However, we recognize that some centers may be incentivized to undertake assessment to gain formal recognition of their services. The process for recognition will be informed by the current user-testing being undertaken in 10 countries over 5 continents and will draw upon and align with existing initiatives for accreditation of stroke and rehabilitation services, such as the WSO’s stroke center accreditation, Canada’s Stroke Distinction programme, and the Commission on Accreditation of Rehabilitation Facilities (CARF). Critically, the implementation of the criteria for CoCE will support improvements in processes that can engender excellence and so will largely complement and enhance, rather than replicate, existing initiatives which typically target specific elements of clinical care^7,8^ or service delivery^18^ Any redundancies identified between these initiatives and our work in the current user testing will be minimized by aligning with, and signposting to, other programs that promote excellence.

We will continue to work closely with stakeholders including patient groups and representatives from clinical centers to finalize a process for accreditation. Accreditation could comprise centers initially self-evaluating, submitting evidence for each criterion and assessment by a team of objective reviewers who visit the center. This could be undertaken by global ISRRA members or alongside national and international groups who already provide accreditation such as the WSO and CARF. Similarly to the WSO accreditation process, the threshold for a rating of overall excellence is likely to necessitate a minimum level of achievement across all indicators but also recognize excellence in individual criterion. Crucially, any formal assessment would provide detailed developmental feedback for each criterion and facilitate partnerships with other global centers to share expertise. The frequency of assessment of CoCE could be linked to performance with outstanding centers being assessed less frequently than developing centers, as exemplified by CARF.

A strength of this work is that a CoCE is considered as a network of linked services across the stroke pathway, rather than being a discrete service offered at 1 site or by 1 organization. This novel approach places the patient’s “journey” through stroke services at the center of these criteria and indicators, and differs from other methods of describing stroke centers by the services delivered at specific sites.^19^ However, we recognize that not all CoCE will have access to the same range of interventions and services as others and this should be explicitly reflected in the application of the criteria and indicators.

The centrality of key stakeholders, including staff, patients, and their carers, in the development of both criteria and indicators is a key element of our work. This provides a more holistic mechanism to reflect and engender excellence than other definitions which typically examine single indicators of clinical services such as staff expertise, care processes, or patient satisfaction.^5,7,20^ Whilst these individual constructs are important and implicitly included in our criteria and indicators, their presence alone is unlikely to ensure excellence; in contrast, by articulating the *processes* that could facilitate clinical excellence, our work demonstrates clear and tangible ideals that centers can aspire to meet. Despite the diversity of the stakeholders included in the work presented here, it is recognized that not all groups were represented, including managers and administrators of healthcare facilities, policy makers, and other clinicians who are involved in stroke rehabilitation, such as neuropsychologists.

Perhaps unsurprisingly, the criterion ranked as most important to clinical excellence was related to providing optimal outcome for patients. Whilst this is often the focus of clinical guidelines, this criterion demonstrated a novel, holistic approach by considering the patient’s and carer’s wellbeing and their perception of their experiences, rather than solely relying on functional outcomes. Our work recognizes the importance of seeking the views of carers which is particularly prescient when communication or cognition deficits after stroke prevents patients articulating their needs. Other criteria, including research culture and leadership were also recognized to be important, yet rarely feature in guidelines or service standards of practice for stroke rehabilitation, attesting to the novelty and value of our work. Recognition of these broader features is important as they influence the standard of clinical care, and so are likely to significantly influence patient experience and outcomes.^21^

The criteria and indicators produced here embody the ethos of ISRRA and complements the vision of the WSO^16^ as they were intentionally developed to be ambitious and globally applicable, regardless of a country’s development or income status, in contrast to other consensus studies in stroke care.^7^ This global focus, gained from using the views of international, clinically focused experts in stroke rehabilitation and several consumer groups, adds to the strength of this work. The authors explicitly recognize that centers will not have the same resources, infrastructure, and workforce as others so they will begin their journey to clinical excellence from different standpoints and follow a different development trajectory. Whilst countries representing over 3.4 billion of the world’s population were included, a limitation of this work is that countries from Central America, Eastern Europe, and parts of Asia, were not represented. This may mean that the resources, practice of healthcare professionals, and the values of patients from these areas, are not fully reflected by the criteria and indicators. Further work could address this by testing the developed criteria and indicators in these areas to examine their suitability and potentially further refine them for these settings. Nonetheless, the global focus of this work ensured that criteria for CoCE were, though ambitious, broadly applicable to high-, middle-, and low-income countries whilst explicitly acknowledging global differences in the provision of stroke services.^22^ This enables the indicators to be used to transform world-wide stroke care by supporting the stepwise development of clinically excellent stroke centers, sharing learning and facilitating formation of important global partnerships between centers and individuals.

## Conclusions

This work presents the development of criteria and measurable indicators for CoCE in stroke recovery and rehabilitation. It provides an important contribution to understanding how excellence in clinical centers can be defined and articulated. This will enable centers, irrespective of their location or resources, to benchmark and develop their services to improve stroke recovery and rehabilitation. We understand that there are already different quality certifications for stroke services but believe that our criteria and indicators for CoCE provide a novel, complementary, and comprehensive vision of the healthcare process for patients who survive stroke and those that care for them, as well as the processes of the clinical team and the leadership of the organization necessary to achieve the best outcomes.

It is recognized that until the indicators are utilized by stroke centers, their practical capacity to support organizations to become clinically excellent remains unproven. Further work is already underway to understand how the indicators can be implemented by 14 international centers. Whilst ranking centers on their performance was not the primary focus of this work, the possibility of being recognized as providing clinically excellent services after stroke is likely to attract clinical centers that wish to establish themselves as leaders in the field, as well as those who wish to develop their services. This encourages the national and international collaborations explicitly included in our criteria for CoCE and facilitates global centers to work together to improve services. If implemented globally, these criteria may herald a new dawn in the delivery of clinically excellent stroke recovery and rehabilitation, realizing ISRRA’s ambition to bring about major breakthroughs for people living with stroke.

## Supplemental Material

sj-docx-1-nnr-10.1177_15459683231222026 – Supplemental material for Criteria and Indicators for Centers of Clinical Excellence in Stroke Recovery and Rehabilitation: A Global Consensus Facilitated by ISRRA Click here for additional data file. Supplemental material, sj-docx-1-nnr-10.1177_15459683231222026 for Criteria and Indicators for Centers of Clinical Excellence in Stroke Recovery and Rehabilitation: A Global Consensus Facilitated by ISRRA by Rachel C. Stockley, Marion F. Walker, Margit Alt Murphy, Noor Azah Abd Aziz, Philemon Amooba, Leonid Churliov, Amanda Farrin, Natalie A. Fini, Emma Ghaziani, Erin Godecke, Tania Gutierrez-Panchana, Jie Jia, Thoshenthri Kandasamy, Patrice Lindsay, John Solomon, Vincent Thijs, Tierney Tindall, Donna C. Tippett, Caroline Watkins and Elizabeth Lynch in Neurorehabilitation and Neural Repair
