# Supplementary material for: Criteria and Indicators for Centers of Clinical Excellence in Stroke Recovery and Rehabilitation: A Global Consensus Facilitated by ISRRA
Source: Neurorehabil Neural Repair. 2024 Jan 11;38(2):87–98. doi: 10.1177/15459683231222026 (PMC10874109; doi:10.1177/15459683231222026)
Supplement: sj-docx-1-nnr-10.1177_15459683231222026 – Supplemental material for Criteria and Indicators for Centers of Clinical Excellence in Stroke Recovery and Rehabilitation: A Global Consensus Facilitated by ISRRA [file sj-docx-1-nnr-10.1177_15459683231222026.docx]

Supplementary material 1 – Surveys

**SURVEY 1**

- **ISRRA Milestone 6**

The key objective of our working group is to define and develop the criteria for aspirational Clinical Centres of Excellence for Stroke Rehabilitation. This survey is the first part of our agreed methodology in gathering expert views. Please answer ALL the questions below.  There are no right or wrong answers so please share your own personal views with us.

- Question 1) In your view, what would be the main purpose of designating a specific rehabilitation centre as a Clinical Centre of Stroke Rehabilitation Excellence?

- Question 2) What can we learn from other fields who have developed Centres of Clinical Excellence?

- Question 3) What is the role of the voluntary sector in stroke rehabilitation in your health care system? If it does not exist in your country how could you see it contributing to a Centre of Clinical Excellence in Stroke Rehabilitation?

- Based on our discussions to date we have identified 6 fundamental dimensions for a Centre of Clinical Excellence:

1. Centres who focus on delivery of optimal outcomes for people living with stroke across the whole pathway

2. Strong integrated leadership across all areas of the pathway

3. Networking (locally, nationally and internationally) for consultations/sharing information which advances best practice

4. Interdisciplinary working in all areas across the pathway.

5. Research and Innovation that moves all areas of stroke rehabilitation forward

6. Education of patients, carers and staff

- Question 4) Do you agree with these six dimensions?

- Question 5) Have we missed an important dimension? Please tell us anything more you would like to see included in our list of dimensions.

Please differentiate between a truly new additional dimension currently not included on our list and additional detail to the core dimensions listed above (i.e. something that may contribute to a more comprehensive list of dimensions listed in Qu 4)

**SURVEY 2**

**ISRRA Milestone 6 - Survey**

Thank you for your valuable contribution to the recent survey **ranking the agreed key dimensions (criteria) of Centres of Clinical Excellence for Stroke Rehabilitation and Recovery**. We have now collated your responses. The dimension rankings have been analysed and whilst there was clear ranking on some of the dimensions there were three ties with equal ranking.

To now reach a consensus on the three dimensions that were equally ranked, please find the remaining three to be **ranked from 1-3 in order of importance,** **with 1 being the most important**. Please (tick/cross) each value 1, 2 or 3 only once.*

| Key dimensions / criteria * | 1 (most important) | 2 | 3 (least important) |
| --- | --- | --- | --- |
| Centres of Clinical Excellence facilitate interdisciplinary working and patient and carer involvement in all areas across the pathway |  |  |  |
| Centres of Clinical Excellence have strong integrated leadership and patient influence across all areas of the pathway |  |  |  |
| Centres of Clinical Excellence continually network with health care professionals, and patients and carers (locally, nationally and internationally) for consultations and sharing information which advances best practice |  |  |  |

Completed by:

Date:

Please return your completed form to …………………………………….

Bottom of Form

Top of Form

Bottom of Form

**Centre of Clinical Excellence in Stroke Rehabilitation and Recovery – Survey 4**

In stage one of this work, our expert working group of international researchers and consumers bodies identified seven defining criteria of aspirational Centres of Clinical Excellence in stroke rehabilitation and recovery. Following this, the expert group will need to develop key performance indicators for each criterion that centres can use to benchmark where they are in achieving excellence, and identify gaps to improve their services to become a centre of excellence.

For this next stage of work, we are seeking to gather information on what each of the criteria mean in practical terms and how they could be measured. At this stage, we are looking to gather as many views as possible, so please do not limit any of your responses, the more detail and alternative suggestions you can provide, the better.

The seven criteria are listed below. Please read each criterion and then answer both questions using the free text boxes. Please be free and creative in your responses – consider this a brainstorming exercise.

1. Centres of Clinical Excellence in Stroke Rehabilitation and Recovery deliver outstanding rehabilitation to ensure optimal outcome (health, social and wellbeing) for people living with stroke.

- *What are the key elements that define optimal outcome at a clinical centre in stroke rehabilitation and recovery?*
- *In your opinion, what are the factors that*
  1. *Assist in achieving optimal outcome at a clinical centre?*
  2. *Hinders a clinical centre from achieving optimal outcome?*

1. Centres of Clinical Excellence in Stroke Rehabilitation and Recovery have a strongly developed research culture, demonstrated by proactive national and international research collaborations and translation of research into best clinical practice.

- *What are the key elements that define a strongly developed research culture at a clinical centre in stroke rehabilitation and recovery?*
- *In your opinion, what are the factors that*
  1. *Assist in developing a strong research culture?*
  2. *Hinders the development of research culture?*

1. Centres of Clinical Excellence in Stroke Rehabilitation and Recovery ensure interprofessional working and person-centred rehabilitation where colleagues, persons with stroke and carers work together towards a common goal.

- *What are the key elements of excellent interprofessional relationships within a clinical centre in stroke rehabilitation and recovery?*
- *In your opinion, what are the factors that*
  1. *Promote interprofessional relationship?*
  2. *Hinders interprofessional relationship?*

1. Centres of Clinical Excellence in Stroke Rehabilitation and Recovery exchange new knowledge and actively promote mentorship with National/ International colleagues and people living with stroke to advance best practice.

- *What are the key elements involved in the exchange of knowledge and the promotion of mentorship?*
- *In your opinion, what are the factors that*
  1. *Encourage the exchange of knowledge at a clinical centre?*
  2. *Hinders the exchange of knowledge at clinical centre?*

1. Centres of Clinical Excellence in Stroke Rehabilitation and Recovery have a shared strong ethical and value-based leadership, that inspires, motivates and rives forward successful rehabilitation.

- *What are the key elements that promote excellent leadership at a clinical centre in stroke rehabilitation and recovery?*
- *In your opinion, what are the factors that*
  1. *Assist in developing excellent leadership skills?*
  2. *Hinders the development of excellent leadership skills?*

1. Centres of Clinical Excellence in Stroke Rehabilitation and Recovery use their specialist knowledge to provide continuous high-quality education to people with stroke, carers, staff and the general public.

- *What are the key elements of the provision of high quality education at a clinical centre in stroke rehabilitation and recovery?*
- *In your opinion, what are the factors that*
  1. *Promote the provision of the high quality education?*
  2. *Hinders the provision of high quality education?*

1. Centres of Clinical Excellence in Stroke Rehabilitation and Recovery advocate and promote equitable access and optimal delivery of stroke rehabilitation services and funding for innovative research.

- *What are the key elements of advocacy and the promotion of equitable access at a clinical centre in stroke rehabilitation and recovery?*
- *In your opinion, what are the factors that*
  1. *Assist in promoting advocacy and developing equitable access?*
  2. *Hinders advocacy and development of equitable access?*

**Centre of Clinical Excellence in Stroke Rehabilitation and Recovery Survey 5**

Please tell us anything that is unclear, missing or requires further detail for the criteria and measurable indicators.

**1 : Centres of Clinical Excellence in Stroke Rehabilitation and Recovery deliver outstanding rehabilitation to ensure optimal outcome (health, social and wellbeing) for people living with stroke**

1. Optimal outcomes
   1. Patient outcomes
      1. Clinical/physiological measures
      2. Patient reported outcomes
      3. Patient reported experience
      4. Self-management skills
   2. Carer outcomes
      1. Carer reported outcomes
      2. Carer reported experience
      3. Carer self-management skills
   3. Service outcomes
2. Deliver outstanding rehabilitation
   1. Assessment of rehabilitation requirements
      1. Comprehensive/holistic assessment
      2. Ongoing assessment at regular time points
   2. Rehabilitation interventions
      1. Evidence-based
         1. Time of commencement
         2. Duration
         3. Dose
      2. Addresses person’s goals (tailored rehabilitation)
      3. Integrated delivery (minimise duplication between professionals/services)
   3. Coordinated ongoing care and support

**2: Centres of Clinical Excellence in Stroke Rehabilitation and Recovery have a strongly developed research culture, demonstrated by proactive national and international research collaborations and translation of research into best clinical practice**

1. Organisational processes and systems
   1. Research elements in all job descriptions role profiles
   2. Organised initiatives to support positive research culture
      1. Regular research activities for all staff eg journal clibs, training or attending conferences
      2. Embedded quality improvement program
         1. Regular collection of outcome data (for Criterion 1)
   3. Infrastructure and resources to support research activity
      1. Allocated research time
      2. Systems to support high quality data collection
   4. A recognised pathway to implement research into practice
2. Formalised links with external agencies
   1. Links with universities
   2. Research collaborations with other national and international centres
3. Staff expertise and culture
   1. Leading research, applying for and winning research funding
   2. Research leadership from multiple professional groups, not just medical
   3. Broad methodological research knowledge across staff base (or access to skills/knowledge)

**3. Centres of Clinical Excellence in Stroke Rehabilitation and Recovery ensure inter-professional working and person-centred rehabilitation where colleagues, persons with stroke and carers work together towards a common goal.**

1. Organisations and systems to proactively support patient and family involvement in rehabilitation journey
   1. Information provided routinely to patients and family about rehabilitation process and rehabilitation team
   2. Collaborative goal setting process (goals agreed upon by team, patient, family)
   3. Regular opportunities between team, patient and family for 2-way information exchange
   4. Shared decision-making
   5. Virtual communication available when indicated (eg lockdowns, supporting remote services)
   6. Processes to identify all key stakeholders in stroke rehabilitation within and beyond the centre
   7. Culturally safe care provision
2. Systems to support coordinated interdisciplinary teamwork
   1. Regular opportunities for rehabilitation team to collaboratively review patient goals, progress and plans
   2. Input from each team member is respected and valued

**4. Centres of Clinical Excellence exchange new knowledge and actively promote mentorship with National/International colleagues and people living with stroke to advance best practice.**

1. Knowledge exchange
   1. Collaborations with external organisations to exchange knowledge about best practice e.g. clinical practice groups, national and international rehabilitation groups
   2. Protected time allocated for knowledge exchange activities eg networking
   3. Opportunities for staff to participate in training using different modalities for knowledge exchange activities e.g TED talk, social media, radio, TV
2. Mentorship
   1. Formal interdisciplinary mentorship program (i.e. allocated mentors and mentees) for **individuals**
   2. Formal mentorship program for **centres**
   3. Investment in mentorship training for mentors
   4. Protected time for mentoring

**5. Centres of Clinical Excellence in Stroke Rehabilitation and Recovery have a shared strong ethical and value-based leadership, that inspires, motivates and drives forward successful rehabilitation.**

1. Development
   1. Rehabilitation workforce development
      1. Commitment to recruitment of the ‘best’ staff
      2. Processes to promote the growth and development of staff
   2. Leadership development
      1. Mechanisms to gain feedback to/about leaders and assess leadership e.g. 360 degree feedback, formal appraisals, open door policies
      2. Investment in training and time to grow leaders (who are open minded, adaptive, inclusive, team focussed, knowledgeable)
      3. Systems to support staff to take up global leadership roles (e.g. editorial boards, committees)
2. Leaders engaging with key stakeholders
   1. Engagement of leadership with patients and carers
   2. Leadership actively promotes delivery of successful rehabilitation
3. National/international leadership
   1. Representation on influential national/international groups and professional bodies

**6. Centres of Clinical Excellence in Stroke Rehabilitation and Recovery use their specialist knowledge to provide continuous high-quality education to people with stroke, carers, staff and the general public (Formal education such as In-house training, Masters Courses, Conference Presentations, Public Lectures etc).**

1. Receiving education
   1. Pathways for staff to gain higher-degree qualifications including Masters and PhD
   2. Onsite educational opportunities eg inhouse training
   3. Support for off-site education eg sponsored workplace visits, conference scholarships, sabbaticals to other centres
2. Delivering education
   1. Delivering conference presentations and in-services to health professionals
   2. Providing education to stroke survivors and carers, and the public

**7. Centres of Clinical Excellence in Stroke Rehabilitation and Recovery advocate and promote equitable access and optimal delivery of stroke rehabilitation services and funding for innovative research**

1. Processes that facilitate ongoing communication with key stakeholders
2. Equitable access of stroke rehabilitation
   1. Systems to promote equitable access
   2. Process to monitor access
   3. Process to improve access if problems identified
3. Regular advocacy and outreach activities
   1. For access to stroke rehabilitation services
   2. For innovative research
